# Building the evidence base for stigma and discrimination-reduction programming in Thailand: development of tools to measure healthcare stigma and discrimination

**DOI:** 10.1186/s12889-017-4172-4

**Published:** 2017-03-11

**Authors:** Kriengkrai Srithanaviboonchai, Melissa Stockton, Nareerut Pudpong, Suwat Chariyalertsak, Phusit Prakongsai, Chonlisa Chariyalertsak, Piyathida Smutraprapoot, Laura Nyblade

**Affiliations:** 1 Research Institute of Health Sciences, Chiang Mai University, 110 Intavaroros Road, Sriphum, Muang, Chiang Mai, 50200 Thailand; 2 Faculty of Medicine, Chiang Mai University, Chiang Mai, Thailand; 3 Research Triangle Institute, International, Research Triangle Park, North Carolina USA; 4 Healthcare Accreditation Institute (Public Organization), Nonthaburi, Thailand; 5 International Health Policy Program, Ministry of Public Health, Nonthaburi, Thailand; 6 Bureau of International Health, Ministry of Public Health, Nonthaburi, Thailand; 7 Chiang Mai Provincial Health Office, Ministry of Public Health, Chiang Mai, Thailand; 8 Bureau of AIDS, Tuberculosis, and Sexually Transmitted Infections, Bangkok Metropolitan Administration, Bangkok, Thailand

## Abstract

**Background:**

HIV-related stigma and discrimination (S&D) are recognized as key impediments to controlling the HIV epidemic. S&D are particularly detrimental within health care settings because people who are at risk of HIV and people living with HIV (PLHIV) must seek services from health care facilities. Standardized tools and monitoring systems are needed to inform S&D reduction efforts, measure progress, and monitor trends. This article describes the processes followed to adapt and refine a standardized global health facility staff S&D questionnaire for the context of Thailand and develop a similar questionnaire measuring health facility stigma experienced by PLHIV. Both questionnaires are currently being used for the routine monitoring of HIV-related S&D in the Thai healthcare system.

**Methods:**

The questionnaires were adapted through a series of consultative meetings, pre-testing, and revision. The revised questionnaires then underwent field testing, and the data and field experiences were analyzed.

**Results:**

Two brief questionnaires were finalized and are now being used by the Department of Disease Control to collect national routine data for monitoring health facility S&D: 1) a health facility staff questionnaire that collects data on key drivers of S&D in health facilities (i.e., fear of HIV infection, attitudes toward PLHIV and key populations, and health facility policy and environment) and observed enacted stigma and 2) a brief PLHIV questionnaire that captures data on experienced discriminatory practices at health care facilities.

**Conclusions:**

This effort provides an example of how a country can adapt global S&D measurement tools to a local context for use in national routine monitoring. Such data helps to strengthen the national response to HIV through the provision of evidence to shape S&D-reduction programming.

**Electronic supplementary material:**

The online version of this article (doi:10.1186/s12889-017-4172-4) contains supplementary material, which is available to authorized users.

## Background

Thailand is globally recognized for its achievements in controlling the HIV epidemic and establishing quality care and treatment programs for people living with HIV (PLHIV) [1]. However, despite continued HIV prevention efforts and the provision of free and universal access to antiretroviral treatment (ART), AIDS-attributed deaths have remained steady, and the average CD4 count at ART initiation remains low [2]. Additionally, warning signs suggest that Thailand may face another wave of the HIV epidemic; indeed, new infections among key populations (KPs), especially men who have sex with men, are rising [3].

Globally, the detrimental effect of stigma and discrimination (S&D) on HIV prevention and, ultimately, each step in the HIV care continuum—testing, linkage to care, retention in care, adherence, and viral suppression—is increasingly well documented [4–10]. Although S&D occur at each socio-ecological level of society, HIV-related S&D in health facilities is particularly harmful to health and wellbeing and has been documented in multiple studies worldwide [11–15]. Additionally, growing evidence indicates that S&D are key factors hampering an effective HIV response in Thailand by undermining HIV testing, treatment, and retention [16]. The 2009–2010 “Index of Stigma and Discrimination against People living with HIV/AIDS in Thailand” study confirmed the prevalence of many forms of S&D [17], and other studies in Thailand have also demonstrated the negative influence of S&D on prevention and treatment. A study among men who have sex with men and transgender persons found that HIV-related S&D were inversely associated with the intention to test for HIV and rectal microbicide acceptability [18], and a survey of PLHIV linked HIV-related stigma with low adherence to ART [19]. Furthermore, a qualitative study found that perceived stigma, shame, and fear of rejection were key barriers to HIV disclosure among PLHIV [20]. Stigma toward PLHIV exists throughout Thai society, as reflected by the results of the recent National Health Examination Survey of Thai households: 51% of respondents reported that they would not buy fresh vegetables from a shopkeeper or vendor if they knew that the seller had HIV, and 22% thought that children living with HIV should not attend school with HIV-negative children [21].

Given this situation, the 2014–2016 Thailand National AIDS strategy set S&D reduction as a key goal and established a target of halving the prevalence of discrimination against PLHIV and other KPs by the end of 2016 relative to the level in 2012 [22]. As part of achieving this target, the National AIDS strategy also recognizes that routine S&D data collection is important to be able to track progress and design effective S&D-reduction programs. In response, the National AIDS Management Center, which is the government body under the Department of Disease Control in the Ministry of Public Health tasked with coordinating national data collection for HIV response, coordinated a study team of academics from the International Health Policy Program and Chiang Mai University and the public health staff from the two study sites—Chiang Mai Provincial Health Office and Bangkok Metropolitan Administration—to adapt and pilot a global standardized tool to measure health facility S&D to the Thai context [23].

The global tool [24] was created through an effort to create a brief enough standardized questionnaire that would be feasible to implement in programmatic applications (e.g. routine monitoring and/or evaluation of country-level or health facility-level activities), but still capture the essential domains of stigma within health facilities. This tool was developed through a multi-step process. Global stigma measurement experts reviewed existing validated tools and designed a combined shortened questionnaire to cover the key HIV stigma domains shown to be important for stigma-reduction programming in health facilities. This questionnaire was then field-tested in six diverse country settings. Resulting data was analyzed across sites using exploratory factor analysis, principle component analysis and combined with qualitative data from the field administration experience. Principal investigators across sites met in person to review all evidence and come to consensus on what items to retain and which ones to remove. The final global tool not only measures manifestations of stigma but also the following key actionable drivers of stigma: fear of HIV infection, attitudes toward PLHIV and KPs, and health facility policies and environment [24]. These stigma drivers are modifiable and can be addressed through targeted stigma-reduction interventions. The global measurement tool was also designed to capture the perspectives of all cadres of healthcare facility staff from clinical care providers to non-medical professionals.

This paper describes the process of adapting the standardized global S&D health facility questionnaire for the Thai context and developing a new tool for measuring the experience of health facility-related S&D among PLHIV in Thailand.

## Methods

### Questionnaire adaptation and development process

**Fig. 1:**
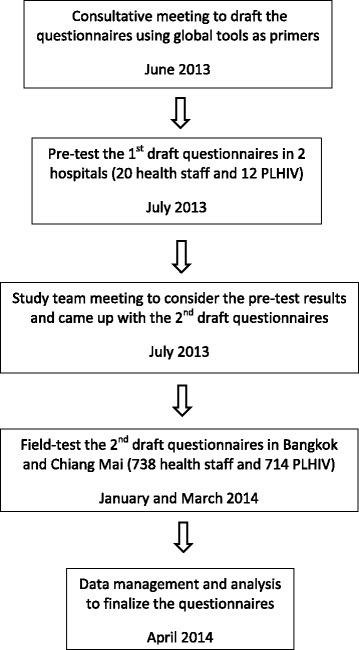
Process and timeline for questionnaire development

The primary purpose of this exercise was to develop brief standardized questionnaires that capture key HIV-related S&D domains in Thai healthcare facility settings for use in a national monitoring system. Figure 1 depicts the five key steps of the process used; each step is described in more detail below.

### Consultative meeting to adapt and draft the questionnaires

To kick-off the process, a three-day consultative meeting was convened to adapt the two questionnaires (health facility staff and PLHIV). Participants included multiple stakeholders from civil society (PLHIV, KPs, and non-governmental organizations), the donors (the International Labor Organization] and United States Agency for International Development), and other international organizations (the Joint United Nations Programme on HIV/AIDS, United Nations Development Programme, United Nations Population Fund, and United Nations Children’s Fund). Using the global health facility survey questionnaire [23] as the base, participants added and adapted content until consensus that the questionnaire covered all necessary topics and adequately suited the Thai context was reached. The PLHIV questionnaire was constructed in parallel by adopting questions designed to capture stigma in health facilities from the 2009 Thai PLHIV Stigma Index Survey [25].

The first draft of the Thai health facility staff questionnaire contained 59 questions and six sections: background information, infection control, health facility environment, health facility policies, attitudes toward PLHIV and KPs, and a special module on stigma toward pregnant women living with HIV for staff who work with pregnant women. The questionnaire was significantly longer than the global questionnaire because questions were added to capture additional dimensions that the group felt might be key in the Thai context. For example, specific questions pertaining to KPs who are particularly vulnerable to HIV and S&D in the Thai setting were included. Because participant literacy was not a concern, it was decided that the health facility staff questionnaire would be self-administered to reduce response bias.

The first draft of the PLHIV questionnaire contained 31 questions and five sections: demographic characteristics and history of HIV infection and treatment, experience of receiving health services in the context of discrimination, internalized stigma, disclosure of HIV status, and discrimination related to reproductive health. A sixth section contained questions related to pregnancy that were asked only of women living with HIV. Questions about discriminatory practices that may be specific to living with HIV in Thailand addressed topics such as being asked to place a hospital robe in an area or basket specifically designated for HIV or being advised to avoid becoming pregnant (e.g., being told “Don’t sin anymore”).

The meeting participants also agreed that capturing information on sexual orientation, gender identity, and other KP status (i.e., sex workers, migrants, and persons who inject drugs) would be important. Given the sensitive nature of this information, this question was asked at the end of the questionnaire. Respondents were given the option to respond directly to the interviewer or to read and complete the question themselves. The PLHIV questionnaire was designed to be administered through face-to-face interviews because the research team anticipated some challenges with literacy amongst the potential respondents.

### Questionnaire pre-testing

The first-draft questionnaires were pre-tested in two government hospitals in the provinces of Nonthaburi and Lampoon, which are fairly similar to the provinces in which the future field testing would occur in terms of their language, culture, and environment. The health facility staff questionnaire was piloted among purposive samples of 10 participants in each hospital: five health staff who were directly involved in caring for PLHIV (i.e., a physician, counselor nurse, ART clinic nurse, tuberculosis clinic nurse, and sexually transmitted infection clinic nurse) and five heath staff whose work did not directly involve caring for PLHIV. These participants then participated in two focus group discussions (one at each hospital) to provide feedback on the questionnaire. The first-draft PLHIV questionnaire was piloted with six PLHIV at each hospital. After the interviews, the respondents were asked to provide feedback on the content and language of the questionnaires individually.

### Study team meeting to consider the pre-test results and respondent feedback

The study team revised the questionnaires to address the issues raised by the pre-test. Most health staff respondents found the questionnaire to be understandable but recommended that the background and purpose of the survey be explained more clearly at the outset. They also suggested changes to the wording and content of some questions. For example, they noted that questions on concerns relating to touching the clothing or bedding of PLHIV needed to specify that the answer should be limited to the scope of work in the hospital and should not include behavior at home or outside the hospital. They also found the KP section quite lengthy and repetitive and indicated that it be shortened or re-formatted into a table. PLHIV respondents identified several confusingly worded questions and suggested alternative language. For example, they noted that questions asking about experiences accessing health services needed to clarify that “using health services” meant “accessing care from ‘any health facility,’” not just the one they were being interviewed at. Overall, revisions were minimal to both questionnaires and mainly related to the format, language, and flow of the questions. Tables 3 and 4 provide details about the second drafts of both questionnaires that were then field tested.

### Questionnaire field testing

The second-draft questionnaires were field tested in selected hospitals in Bangkok and Chiang Mai provinces. Bangkok was chosen to represent an urban setting, whereas Chiang Mai represents a rural setting, with most field-testing sites being community hospitals located far from Chiang Mai city.

#### Sample size

The target sample size was 350 health facility staff and PLHIV in each site, with 300 PLHIV respondents recruited through hospitals and 50 from PLHIV networks (Tables 1 and 2).

**Table 1:** Number of health staff respondents, by study site and hospital type

Hospital type	Bangkok		Chiang Mai	
	no. of hospitals	no. of participants	no. of hospitals	no. of participants
Government	11	289	15	304
Private	3	90	3	55
Total	14	379	18	359

**Table 2:** Number of PLHIV respondents, by study site and location

Hospital type	Bangkok		Chiang Mai	
	no. of locations	no. of locations	no. of locations	no. of locations
Government hospital	6	210	6	230
Private hospital	3	105	2	69
PLHIV network	6	50	3	50
Total	15	365	11	349

#### Sampling strategy

Only facilities with ART clinics were invited to participate. In Bangkok, this included all government hospitals under the Bangkok Metropolitan Administration and Department of Medical Services and purposively selected private hospitals. In Chiang Mai, this was all community hospitals under the Ministry of Public Health, which were then stratified by number of PLHIV clients as follows: small (<300; 11 hospitals), medium (300–400; seven hospitals), and large (>400; six hospitals). Five hospitals from each stratum were purposively selected. Three private hospitals in Chiang Mai were also purposively approached. In total, 32 hospitals in both locations agreed to participate: 26 government facilities and six private facilities.

Eligible medical and non-medical staff were categorized into two groups: Group 1 worked in wards where PLHIV received care (e.g., HIV counseling, ART, Tuberculosis, Sexually Transmitted Infection, and antenatal care clinics), and Group 2 worked in other hospital units. In Bangkok and Chiang Mai, all Group 1 staff were approached for an interview (5–10 people per hospital). For Group 2, the goal was 15–20 staff per hospital in Chiang Mai and 20–25 staff per hospital in Bangkok. Staff without direct patient contact, such as administrative staff, accountants, bookkeepers, and engineering/maintenance staff, were excluded.

Because the hospitals in Chiang Mai were small, the research team was able to obtain a list of all staff names organized by profession and department from which to draw the sample. A systematic random sampling technique was used to choose the Group 2 staff from each hospital until the desired sample size was achieved. In Bangkok, complete lists of all staff working in the hospitals were unavailable because of the complex structure of the hospitals’ administration systems. Therefore, the research team approached major departments within each hospital to determine the total numbers of department staff by profession. These numbers were then used to determine the number of staff by profession to be interviewed in each department. The data collector then approached the departments with the number and list of staff types to be collected and invited all staff in the targeted categories who were working that day to complete the questionnaire until the target number for each staff type in each department was reached.

PLHIV respondents were recruited at a subset of conveniently selected health facilities: six government hospitals and three private hospitals in Bangkok and six government hospitals (two hospitals representing each size category) and two private hospitals in Chiang Mai. To achieve the target samples of PLHIV from the clinics, 30–45 PLHIV were interviewed at each hospital in both locations. Potential patient participants were approached privately by ART clinic staff during their scheduled appointments, provided a brief overview of the research study, and then invited to participate in the study. Interested participants were introduced to an interviewer in a private room. A consecutive sampling technique was used until the target sample size was achieved. A convenience sample of 10–15 PLHIV respondents were recruited from six PLHIV networks in Bangkok and three PLHIV networks in Chiang Mai. Eligible PLHIV participants were 18 years or older and were either currently accessing care from the selected facility or referred by associated networks.

#### Data collection process

The study received ethical approval from the Institute for the Development of Human Research Protections, Bangkok Metropolitan Administration three individual hospitals in Bangkok, and Chiang Mai provincial health office.

Typically, the study team gathered potential health staff respondents in a conference room and shared the study objectives, participation benefits and risks, and time required to participate and stressed that participation was voluntary and that responses would be confidential. Interested participants provided signed inform consent before filling out the questionnaire. Each respondent received a small gift (worth US$6) to compensate them for their time.

PLHIV at health facilities who sought out the interviewer after learning about the study from a health provider were informed about the purpose of the study and the risks and benefits of participation and were assured that participation was entirely voluntary, that the interview could be stopped at any time they wished, that their answers were confidential, and that participation would not affect future services received at the facility. If the participant still wanted to participate and provided written informed consent, a face-to-face interview was conducted in a private room. For PLHIV recruited through a network, the network coordinators were responsible for providing basic information about the study and inviting members to participate. For those who chose to participate, a process similar to that described above was followed, including the collection of signed informed consent. Trained interviewers conducted all interviews and were neither employed by any of the participating health facilities nor members of any of the partnering PLHIV networks. The respondents in Bangkok received 500 baht (~US$14), and the respondents in Chiang Mai received 300 Baht (~US$9) to compensate them for their time and traveling costs.

#### Data management and initial analysis

Data from the field test were entered into Excel, transferred into STATA (version 13), and cleaned. Preliminary data analysis was conducted looking at basic frequencies, cross tabs, and factor analysis in preparation for a full-team data analysis workshop.

### Data analysis workshop

A five-day data analysis workshop was held to consider the field-implementation experience and conduct joint data analysis. Participants included the study team members and an international expert who was involved in the development of the global tool. Key feedback from the field implementation was that both questionnaires were too long. Additionally, the questionnaires needed to be shortened to be feasible for used by the Ministry of Public Health to collect routine S&D monitoring data. Thus, the overarching goal of the analysis was to reduce the questionnaires without sacrificing their ability to comprehensively measure key drivers and manifestations of stigma.

The field-testing implementation experience was used to evaluate the questionnaire items in terms of the following criteria: *“comprehension”* (Did the respondents easily understand the question? Did they have to ask for clarification? Did interviewers have to rephrase questions to make them more understandable? If so, was translation the root of the issue or was the question asked in a way that did not convey the meaning of what we were trying to capture?), *“sensitivity”* (Did the question make the respondent uncomfortable? Did this lead to a refusal to answer?), and *“flow and length of the questionnaire”* (Were there any issues with skip patterns? Was it difficult for respondents to follow the flow of the questionnaire? Did respondents complain about the length of the questionnaire?).

*“Item performance”* was examined by assessing each individual item and sets of items grouped together based on the following criteria: variability, missing or misplaced responses, relevance, and criterion validity. The items were then further assessed using count variables, cross-tabulations, and universality. The details of the resulting questionnaires are described below.

## Results

### Final brief health staff questionnaire [see Additional file 1 for details]

This questionnaire consists of 14 questions containing 26 items. Although it is significantly shorter than the field-test version, the final questionnaire still captures every domain included in prior versions, except for the module on pregnant women living with HIV. This section was deleted because it was not relevant for most of the sampled health staff, however, an attitudinal question regarding childbearing among PLHIV was retained as part of the attitudes section. Table 3 presents the number of questions/items in each section of the global questionnaire, the Thai field-test questionnaire, and the final Thai brief questionnaire. Note, the differences between the global questionnaire and the final Thai brief questionnaire were minimal (Table 3).

**Table 3:** Results of health facility staff questionnaire development

Section		Issues questioned	Global questionnaire		Field-test questionnaire		Final brief questionnaire	
			No. of question	No. of item	No. of question	No. of item	No. of question	No. of item
Background information		Demographics	2	2	3	3	-	-
		Jobs related	4	4	4	9	1	1
		Training on S&D	1	1	1	8	2	3
		Total	7	7	8	20	3	4
Key drivers of stigma	Infection control	Fear of HIV infection	1	4	1	4	1	3
		Avoidance Behavior driven by fear	1	4	1	3	1	2
		Supplies	-	-	1	1	-	-
		Total	2	8	3	8	2	5
	Health facility policies	HIV testing	1	1	1	1	1	1
		Punishment for discrimination	1	1	1	1	1	1
		Supplies and standard procedures	1	2	1	2	1	1
		Written guideline on S&D	1	1	1	1	1	1
		Not allow PLHIV to be staff	-	-	1	2	-	-
		Total	4	5	5	7	4	4
	Opinions about PLHIV and KP	PLHIV	1	5	1	4	1	4
		Women living with HIV	1	1	2	2	1	1
		KP	3	12	34	59	1	5
		Total	5	18	37	65	3	10
Manifestations of stigma	Enacted stigma	Screening question	1	1	1	1	-	-
		Observed stigma	1	3	1	3	1	2
		Attitude towards PLHIV staff	1	3	2	2	1	1
		Secondary stigma	1	1	1	3	-	-
		Total	4	8	5	9	2	3
Module for stigma towards pregnant women living with HIV		Fear of HIV infection	1	1	1	1	-	-
		Observed stigma	1	5	1	5	-	-
		Attitudes towards HIV positive pregnant women	1	4	1	4	-	-
		Total	3	10	3	10	-	-
Total			25	56	61	119	14	26

### Background information

The number of background information questions was reduced from eight to three in the final brief questionnaire; the retained questions included current position and two questions assessing whether respondents have received specific training in S&D reduction. Questions on age, sex, years of work, and number of HIV patients served were removed given a programmatic decision to target all health facility staff with S&D-reduction activities; as a result, these questions were, therefore, deemed unnecessary.

### Key drivers of stigma

#### Infection control

The number of questions asking about infection control was reduced from three to two in the final brief questionnaire. The first retained question asks about the level of fear of HIV transmission while conducting routine activities in the health setting and has three items: touching the personal belongings of, dressing the wounds of, and drawing blood from PLHIV. These three items were kept because they covered both a range of typical actions that different health staff might engage in and a range of HIV transmission risks. The second retained questioned assess self-reported stigmatizing avoidance behavior driven by transmission fear; this question included two items: wearing double gloves and typically using special precautions for infection control with PLHIV only.

#### Health facility policy

The number of questions asking about health facility policy was reduced from five to four in the final brief questionnaire. Retained questions probed respondents’ views regarding HIV testing with patients’ consent, repercussions for discriminating against PLHIV, the adequacy of supplies to protect health staff from HIV infection, and the existence of written guidelines to protect PLHIV patients from discrimination.

#### Opinions about PLHIV and Attitudes and Behaviors towards KPs

The number of questions asking about attitudes toward PLHIV and KPs was reduced from 37 to only two in the final brief questionnaire. The retained questions were as follows: 1) the field-tested question asking about negative attitudes toward PLHIV and its four items and 2) a question about whether women living with HIV should be allowed to have children. Numerous attitudinal questions relating to KPs were also considered. Given the length constraints (and the fact that each KP question essentially adds multiple questions, one for each KP of interest), the team examined the response to each question and concluded that the most important question to keep was one relating to observed stigma (see the section on enacted stigma below). Indeed, this question returned the highest proportion of responses among the KP-specific questions, and the workshop participants felt that this was the most serious discriminatory action measured in the field test.

### Manifestations of stigma

#### Enacted stigma

The number of questions asking about enacted stigma toward PLHIV was reduced from five to two in the final brief questionnaire. One question addressed observed stigma in the past 12 months with two items: unwillingness to care for and the provision of a reduced quality of care to PLHIV. Observed stigma, rather than the respondents’ behaviors themselves, was asked about because most health staff know that such acts of discrimination are not socially desirable and, thus, would likely under report. A question assessing the level of comfort with working with co-workers who are living with HIV was also retained. The questions on secondary stigma associated with providing care to PLHIV were removed because of lack of variation. Finally, a question asking about observing (in the past 12 months) other health facility staff being unwilling to care for a patient in the past 12 months who was or was thought to be from one of five KP groups (men who have sex with men, transgender women, sex workers, drug users, and migrants) was included in table format.

### The final brief PLHIV questionnaire [see Additional file 2 for details]

The final brief PLHIV questionnaire includes 17 questions with 33 items covering five domains (Table 4).

**Table 4:** Results of PLHIV questionnaire development

Section	Issues questioned	Field-test questionnaire		Final questionnaire	
		No. of question	No. of item	No. of question	No. of item
Background	Demographics	5	6	2	2
	Usage of health services	3	5	3	4
	Gender and KP	2	2	1	1
	Total	10	13	6	7
Health related stigma	Anticipated stigma	3	5	2	6
	Experienced stigma	1	21	1	11
	Internalized stigma	1	9	1	2
	Total	5	35	4	19
Disclosure and confidentiality	Disclosure of HIV status	2	2	1	1
	Confidentiality	1	1	2	2
	Total	3	3	3	3
S&D in relation to reproductive health	Sex and marriage	2	2	1	1
	Having children	4	4	3	3
	Total	6	6	4	4
Experience of stigma among pregnant women	Screening question	1	3	-	-
	Termination of pregnancy	1	2	-	-
	Prevention of mother to child HIV transmission	1	2	-	-
	Disclosure of HIV status	2	2	-	-
	Total	5	9	-	-
Total		29	66	17	33

#### Background information

The number of background information questions was reduced from 10 to six in the final brief questionnaire. The first five questions on age, type of health insurance, duration of using health care services, length of knowing HIV status, and status of ART were placed at the start of the questionnaire. Because of its sensitivity, the question on gender identity (i.e., male, female, or transgender), sexual orientation (i.e., gay, lesbian, or bisexual), and other KP status (i.e., people who inject drugs, sex workers, or migrants) was included at the end of the questionnaire. Respondents could opt to answer this question on their own and seal the questionnaire or have the interviewer check the answers for them. This strategy worked well, with many respondents opting to answer this question on their own.

#### Health-related stigma

This section included questions about anticipated, experienced, and internalized stigma related to the utilization of health services. The number of questions was reduced from five to four in the final brief questionnaire. The first question asked whether anticipated stigma hampered access to care and treatment among PLHIV: *“In the last 12 months, have you avoided going to or delayed going to a health care facility near your home for HIV-specific services or for other general health issues/problems (not specific to HIV illness)?”* A similar question—about ANC and prevention of mother-to-child HIV transmission services—was asked of women who had ever been pregnant while living with HIV. These questions have three response categories that the interviewers can choose from as reasons for the respondent’s avoiding/delaying accessing services: 1) stigma-related reasons (e.g., fear of disclosure of HIV status), 2) stigma-related reasons regarding quality of care (e.g., anticipated unfriendly services), and 3) non-stigma-related reasons (e.g., no transportation or no health insurance). The field-tested ‘experience of stigma among pregnant women’ section was removed to avoid repetition.

Eleven out of 19 field-tested items measuring experienced stigma at health care facilities were retained: denial of care, treatment given on a conditional basis, delay of care, patient’s medical record visibly marked as HIV positive, talked with rudely (e.g., scolded or blamed), poor quality of care, service providers’ avoidance of touching them, and discrimination in an in-patient ward (e.g., bed marked as being HIV positive).

This section also included one self-stigma question with two sub-items that asked about feeling ashamed or guilty for being HIV positive.

#### Disclosure and confidentiality

Three questions regarding disclosure and confidentiality of HIV status were retained in the final PLHIV questionnaire: 1) involuntary disclosure of HIV status by health facility staff to other people without the patient’s consent, 2) respondents’ level of confidence that health providers would keep their HIV status confidential, and 3) non-adherence to ART because of a fear that taking the medication would reveal HIV status to others. The question asking how health care providers knew the respondent was living with HIV was dropped because the disclosure of HIV status to health care providers is generally unavoidable and acceptable in the Thai context.

#### Stigma in relation to reproductive health

The number of questions asked of both male and female respondents about reproductive health was reduced from six to four in the final brief questionnaire. The four retained questions included were the following: ever being advised not to have sex, not to have children, or to terminate a pregnancy, or to have been provided ART under the condition of taking contraception or undergoing sterilization.

## Discussions

The process Thailand used to systematically adapt a global standardized health facility staff S&D questionnaire and develop a PLHIV questionnaire for the routine monitoring of HIV-related S&D provides a roadmap and lessons learned for other countries seeking to measure S&D. The adaptation process in Thailand demonstrates that the standardized global measurement instrument for health facility staff, which was originally tested in six countries with different HIV epidemic, health system, language and cultural contexts, also worked well for Thailand. Most of the global questionnaire was retained in the final brief Thai version, indicating that other countries may be able to adapt the global questionnaire with minimal effort to their local context and monitoring needs.

This process also demonstrated the importance of collecting the patient perspective alongside that of health facility staff. Having both perspectives allows for the triangulation of data by comparing PLHIV reporting experienced stigma in health facilities with health staff reporting observed stigma. Having data from both perspectives can in and of itself provide an important stigma-reduction intervention opportunity. When staff and clients share data from their differing perspectives, this can provide ‘neutral’ evidence to initiate discussions and dialogue on how to change the situation described by the data.

Several key factors contributed to the success of this adaptation process. First was the inclusion of all key stakeholders. Bringing together diverse perspectives and experiences from the outset helped ensure that no significant questions were left out and that the final product would be widely accepted and utilized. Second, a strong partnership between the Ministry of Public Health, which is the authoritative body responsible for all HIV-related activities, and researchers with available time and expertise contributed to the success achieved here. The National AID Management Committee, which is responsible for the national HIV monitoring system, helped coordinate the study greatly facilitating collaboration with the selected health facilities. Lastly, the study was supported through joint funding by the International Labor Organization, United States Agency for International Development, and the U.S. President’s Emergency Plan for AIDS Relief, which allowed this study to receive strong technical assistance from international experts.

The study benefitted in many ways from the input of experts who took part in the development of the global tools. These experts helped ensure that all key concepts and theories were understood by the local teams; that technical terms and their meanings would be consistent with those used in the global tools; that the processes used to develop, test, and refine the tools would be scientifically rigorous; and that all necessary S&D domains would be retained in the final brief questionnaires.

In addition to the questionnaires, the study team also developed a survey guidelines and procedures manual (available in Thai and English) [26]. It provides guidance on sample size calculation techniques, sampling procedures, informed consent procedures, data collection, data entry, and quality assurance. This by-product has been used by provincial public health staff as implementation guidelines and as training material during the establishment of a national S&D monitoring system.

In 2015, the national network of the surveillance system used to monitor HIV-related S&D in Thai health care settings was established under the leadership of the National AIDS Management Committee with technical support from this study’s research team. The network comprises sites in seven provinces, including the two provinces where field-testing was conducted. The first round of routine data collection was successfully implemented by the provincial teams using the tools and methodology developed in this study. A data analysis and result interpretation workshop involving the provincial S&D data collection teams was held at the provincial health office and teams were encouraged to bring the data back to their provinces to use in designing S&D-reduction interventions. At the central level, the results are being used as baseline national estimates and as inputs for designing a national health facility intervention approach, which is currently being pilot tested in government hospitals in four selected provinces. In 2015–2016, twelve provinces outside the nationally supported surveillance network used their own provincial resources to collect S&D data, and at least 7 more provinces will do so in 2017. These actions demonstrate the growing commitment to understanding and addressing S&D across the country.

Data and interventions beyond the health care settings will also be needed to fully and strategically address HIV-related S&D. For Thailand, other activities regarding HIV-related S&D are also planned. To support these activities, questions on attitudes toward PLHIV were included in the recently conducted 5th national health examination survey [21]. This large multi-stage probability sampling survey of the general adult public included the questions necessary to report on the Global AIDS Response Progress Reporting global indicator for discriminatory attitudes toward PLHIV [27]. Using processes similar to those used in this study, another set of brief questions was also developed to monitor the S&D experienced by KPs. The national HIV data committee recently endorsed the incorporation of these questions into the nation’s next round of the Integrated Biological and Behavioral Surveillance survey.

There are some limitations inherent to this study and adaptation process. The tools were tested at only two sites, and thus, the data gathered might not represent the opinions of all health staff and PLHIV in Thailand. The Thai tool was modeled after the standardized global tool that, while evidence-based and programmatically useful, had to allow for variability in sampling methodology and administration. Further statistical analysis to compare the global S&D measurement tool to the Thai measurement tool would have been valuable and could possibly have strengthened the adaptation and refinement process. However, as the Thai questionnaire included only minimal changes from the global questionnaire, formal re-validation was deemed unnecessary. In future adaptation efforts, cross-context validation is advisable should more extensive adaptations be undertaken. Despite these shortcomings, the Thai tool covers each of the stigma domains deemed pertinent to stigma-reduction in healthcare facilities, is practical and feasible to implement and is being used to support stigma measurement, reduction, and monitoring for the Thai national program to reduce S&D. That twelve provinces in Thailand chose to use their own provincial funds to collect S&D data using these tools indicates the usefulness, appropriateness and feasibility of implementation of these tools. Additionally, the lengthy and elaborate process that Thailand went through, while yielding quality results, may not be replicable by other countries because of time and resource constraints. However, in sharing the Thai experience, we hope to provide a model process that can be a flexible and adaptable guide for others and to share an experience demonstrating that the global tool can likely be adapted via a much shorter process than that used by Thailand.

The overall process establishes that S&D can be measured at scale as part of a national HIV response and provide essential data for developing evidence-based stigma-reduction programming for health facilities. The transferability of the measurement tools and approach is currently being demonstrated by the implementation of a similar approach with a much briefer adaptation process in Lao People’s Democratic Republic with technical assistance from this study’s research team. Both processes demonstrate how a global tool for measuring the HIV-related S&D in health care settings can be readily adapted and used for routine monitoring. Given the growing recognition of the severity of S&D in healthcare facilities, global targets have been set to prioritize the provision of stigma-free health services [28, 29]. The tools and processes described here offer an example of how to monitor progress toward meeting those targets and collect data to inform interventions to achieve those targets.

## Conclusions

Measuring S&D for routine national monitoring and stigma-reduction intervention design and evaluation is feasible, as demonstrated by the work performed in Thailand. The experience of adapting a standardized global tool for measuring health facility staff S&D in Thailand provides a roadmap for other countries.

## Additional files

Additional file 1: Final brief health staff questionnaire. (PDF 77 kb)
Additional file 2: The final brief PLHIV questionnaire. (PDF 100 kb)

## Abbreviations

ART: Antiretroviral treatment
KP: Key population
PLHIV: People living with HIV
S&D: Stigma and discrimination
