# Supplementary material for: Building the evidence base for stigma and discrimination-reduction programming in Thailand: development of tools to measure healthcare stigma and discrimination
Source: BMC Public Health. 2017 Mar 11;17:245. doi: 10.1186/s12889-017-4172-4 (PMC5346237; doi:10.1186/s12889-017-4172-4)
Supplement: Additional file 1: — Final brief health staff questionnaire. (PDF 77 kb) [file 12889_2017_4172_MOESM1_ESM.pdf]

1.2 Final: Comprehensive Health Staff Questionnaire (for sentinel surveillance)

Code......................

Health Facility Staff Questionnaire
Interview Date: ____ ____ 2014

Part 1: General/Personal Information

1. What is your current position (only choose one that applies).

- ☐ 1. Physician
- ☐ 2. Dentist
- ☐ 3. Pharmacist
- ☐ 4. Nurse
- ☐ 5. Medical Lab Technician
- ☐ 6. Nurse aide
- ☐ 7. Cashier
- ☐ 8. Receptionist
- ☐ 9. Social Worker Hospital porter
- ☐ 10. Waiter/Waitress (those serve foods to patients)
- ☐ 11. Dental Assistant Counselor
- ☐ 12. Medical Record Staff
- ☐ 13. Translator/Interpreter
- ☐ 14. Support staff to patients
- ☐ 15. Health Education Staff
- ☐ 16. Counselor/Advisor
- ☐ 17. Cleaning Staff/Janitor/Maid
- ☐ 18. Volunteer/PLHIV network Focal Point
- ☐ 19. Other (please specify) ....

Part 2: Infection Control and Prevention

2. How worried would you be about getting HIV infection if you did the followings?

Situation	Not Warried	A Little Worried	Worried	Very Worried	N/A
2.1 Touched the clothing, bedding or belongings of a patient living with HIV or AIDS patient	☐ 0.	☐ 1.	☐ 2.	☐ 3.	☐ 99.
2.2 Dressed the wounds of a patient living with HIV or AIDS patient	☐ 0.	☐ 1.	☐ 2.	☐ 3.	☐ 99.
2.3 Drew blood from a patient living with HIV or AIDS patient	☐ 0.	☐ 1.	☐ 2.	☐ 3.	☐ 99.

3. Do you typically do any of the following measures when providing care or services for a PLHIV or AIDS patient:

Situation	Yes	No	N/A
3.1 Wear double gloves	☐ 1.	☐ 0.	☐ 99.
3.2 Use any special infection control/prevention measures with PLHIV or AIDS patients that you do not use with other patients.	☐ 1.	☐ 0.	☐ 99.

Part 3: Health Facility Environment

4. In the past one year, how often have you observed the following in your health facility?

Situation	Never	Once or Twice	Several Times	Most of the Time
4.1 Health care workers were unwilling to care for a patient living with or thought to be living with HIV.	☐ 0.	☐ 1.	☐ 2.	☐ 3.
4.2 Health care workers were providing poorer quality of care to a patient living with or thought to be living with HIV than to other patients.	☐ 0.	☐ 1.	☐ 2.	☐ 3.

5. Health care workers in this facility feel uncomfortable to work with co-workers or colleagues, who are living with HIV?
   ☐ 1. Comfortable ☐ 2. A little uncomfortable ☐ 3. Uncomfortable ☐ 4. Very uncomfortable

Part 4: Health Facility Policies

6. In this health facility, it is not acceptable to perform the blood test for HIV without a patient's knowledge or consent.
   ☐ 1. Strongly agree ☐ 2. Agree ☐ 3. Disagree ☐ 4. Strongly disagree

7. In this health facility, I will get in trouble (or have negative impacts on my job) if I discriminate against PLHIV or AIDS Patients.
   ☐ 1. Strongly agree ☐ 2. Agree ☐ 3. Disagree ☐ 4. Strongly disagree

8. There are adequate supplies in this health facility that reduce my risk of becoming infected with HIV.
   ☐ 1. Strongly agree ☐ 2. Agree ☐ 3. Disagree ☐ 4. Strongly disagree

9. This health facility has written guidelines to protect PLHIV or AIDS patients from discrimination.
   ☐ 1.Yes ☐ 0. No ☐ 99. Don't know/uncertain

Part 5: Opinions about PLHIV

10. What is your opinion about the following statements.

Statement	Strongly agree	Agree	Disagree	Strongly Disagree
10.1 Most PLHIV do not care that they could infect other people.	☐ 1.	☐ 2.	☐ 3.	☐ 4.
10.2 PLHIV should be ashamed about their HIV status.	☐ 1.	☐ 2.	☐ 3.	☐ 4.
10.3 People get infected with HIV because they engage in irresponsible/immoral behaviors.	☐ 1.	☐ 2.	☐ 3.	☐ 4.
10.4 A woman who is HIV positive should be sterilized even though she doesn't want to.	☐ 1.	☐ 2.	☐ 3.	☐ 4.

11. Women living with HIV should be allowed to have babies if they wish.

☐ 1. Strongly agree ☐ 2. Agree ☐ 3. Disagree ☐ 4. Strongly disagree

Part 6: Issues related to Key Affected Populations regardless of their HIV status

12. In the past 12 months, how often have you observed health care workers unwilling to care for a patient who is or thought to be:

Group	Never	Once or Twice	Several Times	Most of the Time
12.1 Gay	☐ 0.	☐ 1.	☐ 2.	☐ 3.
12.2 Transgender	☐ 0.	☐ 1.	☐ 2.	☐ 3.
12.3 Sex worker	☐ 0.	☐ 1.	☐ 2.	☐ 3.
12.4 Drug user	☐ 0.	☐ 1.	☐ 2.	☐ 3.
12.5 Migrant	☐ 0.	☐ 1.	☐ 2.	☐ 3.

13. (BASELINE) Since you started working, have you ever received training in S&D reduction?

☐ 1.Yes ☐ 0. No ☐ 99. Don't know/uncertain

14. (ENDLINE) Post-intervention:

14.1 Since you started working, have you ever received training in S&D reduction?

☐ 1.Yes ☐ 0. No ☐ 99. Don't know/uncertain

14.2 Did you ever participate in the (enter name of S&D reduction training) training?

☐ 1.Yes ☐ 0. No ☐ 99. Don't know/uncertain

****
