# Supplementary material for: Building the evidence base for stigma and discrimination-reduction programming in Thailand: development of tools to measure healthcare stigma and discrimination
Source: BMC Public Health. 2017 Mar 11;17:245. doi: 10.1186/s12889-017-4172-4 (PMC5346237; doi:10.1186/s12889-017-4172-4)
Supplement: Additional file 2: — The final brief PLHIV questionnaire. (PDF 100 kb) [file 12889_2017_4172_MOESM2_ESM.pdf]

2.2 Final: Brief PLHIV Questionnaire

Code.......................

PLHIV Interview Qustions	
Interview Date: ____________________	Name of the Interviewer:____________________

Location of Data Collection:

- ☐ 1. MOPH hospital
- ☐ 2. BMA hospital
- ☐ 3. Sex Worker Network
- ☐ 4. Transgender Network
- ☐ 5. Migrant Worker Network
- ☐ 6. Private Hospital
- ☐ 7. MSM Network
- ☐ 8. IDU Network
- ☐ 9. PLHIV Network (not affiliated with hospital)
- ☐ 10. Other Network (please specify).............

Please ask if the respondent <u>has been interviewed</u> in the past (time perios) on a similar topic of stigma and discrimination, if so, the interview should be terminated.

- ☐ 1. Yes → Terminate interview
- ☐ 2. No → Go to complete the consent form.

Part 1: General/Personal Information

1. How old are you currently: _____ years

2. Currently, what type of health insurance do you hold?
   - ☐ 1. Universal coverage
   - ☐ 2. Social security
   - ☐ 3. Civil Servant Medical Benefit
   - ☐ 4. Health card for migrant workers
   - ☐ 5. Don't have any health insurance
   - ☐ 6. Others, please specify......

3. Are you currently receiving services health care services at any health care facility for any reason (HIV or non-HIV related)?"
   - (If one or more years, enter years): Number of years _____ years
   - (if less than 1 year, enter months): Number of months _____ month(s)
   - (If less than 1 month, enter days): Number of days _____ days

4. How long have you known that you are HIV+ (through blood test)
   - (If one or more years, enter years): Number of years _____ years
   - (if less than 1 year, enter months): Number of months _____ month(s)
   - (If less than 1 month, enter days): Number of days _____ days

5. Currently, are you receiving ARV drugs

☐ 1. Yes, currently receiving.

☐ 2. Used to receive but now stopped → skip to question 6

☐ 3. No, never received, because:

  ☐ 3.1 Still under the threshold of recommended guideline → skip to question 6

  ☐ 3.2 Reached the threshold but have not started the process → skip to question 6.

5.1 How long have you received ARV drugs

(If one or more years, enter years): Number of years ____ years

(if less than 1 year, enter months): Number of months ____ month(s)

(If less than 1 month, enter days): Number of days ____ days

Part 2: Experience at a Health Care Facility

6. In the last 12 months (1 year), have you avoided going to or delayed going to a health care facility near your home for HIV-specific services or general health issues/problems (not specific to HIV illness) (i.e. clinic, health care centers, primary care unit, hospitals- both public/private)?

☐ 1. Yes, have avoided

☐ 0. No, never avoided → if female skip to question 7
→ if male skip to question 8

6.1 If yes, what was the reason why you avoided going to the health care facility nearby your home (select all that applies).

(Interviewer: Do not read the options to the respondent. Try to check the options that closely align with the responses made by the respondent and end this question with "Do you have any more reasons to provide?")

☐ 1. Stigma related reasons-fear	☐ 2. Stigma related reasons-quality of service	☐ 3. Non-stigma related reasons
☐ 1.1 Fear of disclosure of HIV status<br>☐ 1.2 I know someone/family at the facility<br>☐ 1.3 Near my work, so colleagues might see me	☐ 2.1 Unfriendly services<br>☐ 2.1.1 Staff talk badly to me because of my HIV status<br>☐ 2.1.2 Made to wail Longer than non-HIV patients/ Put at end of queue<br>☐ 2.1.3 Avoid touching me<br>☐ 2.1.4 Using double gloves<br>☐ 2.1.5 Staff stare at me or gossip about me<br>☐ 2.2. Previous negative experience	☐ 3.1 Inconvenient, too far, no Transport<br>☐ 3.2 No health insurance, high cost<br>☐ 3.3 Poor quality medical care/treatment, examination/diagnostic procedures, quality or selection of medications, provider knowledge, training, experience (but NOT about provider/patient interaction unless due to HIV status), Don't trust provider's medical knowledge<br>☐ 3.4 Wasn't sick enough, don't want treatment in facility, can treat myself

7. (ASK ONLY TO FEMALES) Have you ever been pregnant since learning about your HIV status or learnt about your HIV status while pregnant?

☐ 1. Yes ☐ 0. No, not pregnant since learning HIV positive → skip to question 8

7.1 Have you ever avoided or delayed going to ante-natal care or seeking or adhering to services to prevent transmission of HIV PMTCT services?

☐ 1. Yes ☐ 0. No → skip to question 8

7.2 If yes, what was the reason why you avoided going to the health care facility nearby your home (select all that applies).

(Interviewer: Do not read the options to the respondent. Try to check the options that closely align with the responses made by the respondent and end this question with "Do you have any more reasons to provide?")

☐ 1. Stigma related reasons-fear	☐ 2. Stigma related reasons-quality of service	☐ 3. Non-stigma related reasons
☐ 1.1 Fear that I need to disclose my HIV status<br>☐ 1.2 I know someone/family at the facility<br>☐ 1.3 Near my work, so colleagues might see me<br>☐ 1.4 Fear people will see me and know I am HIV positive	☐ 2.1 Unfriendly services<br>☐ 2.1.1 Staff talk badly to me because I am HIV positive<br>☐ 2.1.2 Made to wait Longer than other patieints /Put at end of queue<br>☐ 2.1.3 Avoid touching me<br>☐ 2.1.4 Using double gloves<br>☐ 2.1.5 Staff stare at me or gossip about me<br>☐ 2.2 Previous negative experience<br>☐ 2.3 Afraid staff have negative attitudes toward PLHIV	☐ 3.1 Inconvenient, too far, no Transport<br>☐ 3.2 No health insurance, high cost<br>☐ 3.3 Poor quality medical care/treatment, examination/diagnostic procedures, quality or selection of medications, provider knowledge, training, experience (but NOT about provider/patient interaction) Don't trust provider's medical knowledge<br>☐ 3.4 Don't want to change contractor/re-register in new place

8. In the past year, have you been to a health care facility?

☐ 1. Yes → proceed to question 8.1

☐ 0. No, never visited any health facility → skip to question 9

8.1 In the past 12 months, have any of the following happened to you in any health care facility because of your HIV status?

Situation	Yes	No	Don't know/Not relevant
8.1.1 Health provider refused to attend to you or you were denied treatment.	☐ 1.	☐ 0.	☐ 99.
8.1.2 You were given a condition to change your behavior prior to receiving treatment (e.g. Stop having sex, selling sex, using drugs, same-sex behavior or begin using contraception.)	☐ 1.	☐ 0.	☐ 99.
8.1.3 Have you ever been told to come back later, put last in queue or made to wait longer than other patients?	☐ 1.	☐ 0.	☐ 99.
8.1.4 Your record was marked as being HIV positive in a way that let people around you know you are living with HIV	☐ 1.	☐ 0.	☐ 99.
8.1.5 Health provider talked badly, scolded or blamed you for having HIV	☐ 1.	☐ 0.	☐ 99.
8.1.6 You received less care/attention than other patients	☐ 1.	☐ 0.	☐ 99.
8.1.7 Health provider avoided touching your body	☐ 1.	☐ 0.	☐ 99.
8.1.8 In the past 1 year, have you been admitted as an in-patient at a hospital?	☐ 1.	☐ 0.	☐ 99.
	↓ to question 8.1.9	↓ skip to question	9
8.1.9 Your bed was marked as being HIV positive in a way that let people around know you are living with HIV.	☐ 1.	☐ 0.	☐ 99.
8.1.10 You had to stay in an area designated only for HIV positive patients or people living with HIV	☐ 1.	☐ 0.	☐ 99.
8.1.11 Health care provider asked you to place your hospital robe in an area/basket specifically designated for HIV positive patients due to your HIV status.	☐ 1.	☐ 0.	☐ 99.

9. Have you ever decided not to go to a health facility because of the following:

Situation	Yes	No	Don't know
9.1 Feeling ashamed of your HIV status.	☐ 1.	☐ 0.	☐ 99
9.2 Being afraid that health facility staff will stare or gossip about you.	☐ 1.	☐ 0.	☐ 99
9.3 Feeling guilty about your HIV status.	☐ 1.	☐ 0.	☐ 99

10. Have you ever skipped or delayed taking your ARVs because of fear that other people will suspect your HIV status?

☐ 1. Yes ☐ 0. No ☐ 99. Not on ART

Part 3: Disclosure & Confidentiality

11. Has a health care provider ever disclosed your HIV status to other people (including husband/wife, other family members, or other people) without your consent?

☐ 1. Yes ☐ 0. No ☐ 99. Not sure

12. How confidential do you think the medical records relating to your HIV status are

*(**Interviewer**: Read each of the choices/options below so that the respondent can select the one that applies)*

☐ 1. I am sure that my medical records will be kept completely confidential.

☐ 2. I am not sure if my medical records are confidential.

☐ 3. I feel that my medical records are not being kept confidential at all.

Part 4: Having Children and Reproductive Health

Since you were diagnosed as HIV positive, have any of the incidents described below happened to you?

*(**Interviewer**: Ask the questions below without considering the gender of the respondent-all questions can be answered by all genders).*

13. Has a healthcare provider ever advised you "not to have sex" because of your HIV status?

☐ 1. Yes, in the past year ☐ 2. Yes, over 1 year ago ☐ 0. Never ☐ 99. N/A

14. Has a healthcare provider ever advised you not to have children since you were diagnosed as HIV positive?

☐ 1. Yes, in the past year ☐ 2. Yes, over 1 year ago ☐ 0. Never ☐ 99. N/A

15. You were told that you can receive antiretroviral drugs only if you use contraception, including sterilization?

☐ 1. Yes, in the past year ☐ 2. Yes, over 1 year ago ☐ 0. Never ☐ 99. N/A

16. Have you / your partner (for males) ever been advised or coerced to terminate any pregnancy due to your / your partner's (for males) HIV status?

☐ 1. Yes, in the past year ☐ 2. Yes, over 1 year ago ☐ 0. Never ☐ 99. N/A

Interviewer: check the completeness of the questionnaire before going to the last section (Part 5 in the next page)

Part 5: Gender and Diversity

Interviewer to read to the respondent:

"The final question is relatively sensitive. The research team is requesting your kind cooperation in responding to this question. Your answer will be very important and useful for improving health care services that are friendly and equal for everybody. Please note that your response is voluntary, and therefore, you can choose to respond or not to. You can also choose to have me read out the question for you OR you can read the question yourself and respond independently.

(Interviewer: please select the box that applies)

- ☐ 1. Interviewer can read and answer for me.
- ☐ 2. I want to answer on my own (thereafter, seals the questionnaire and drops in a box)

17. Which category do you think you belong in the following groups? (Select all that applies):

- ☐ 1. Male
- ☐ 2. Female
- ☐ 3. Transgender
- ☐ 4. MSM/Gay
- ☐ 5. Lesbian
- ☐ 6. Bisexual
- ☐ 7. Sex worker
- ☐ 8. Person who injects drugs
- ☐ 9. Migrant

****
